# NAFLD as a risk factor for HCC: new rules of engagement?

**DOI:** 10.1007/s12072-016-9731-8

**Published:** 2016-05-05

**Authors:** Ryota Masuzaki, Seth J. Karp, Masao Omata

**Affiliations:** The Transplant Center and Department of Surgery, Vanderbilt University Medical Center, Nashville, TN USA; Department of Gastroenterology, Yamanashi Prefectural Central Hospital, Kofu, Yamanashi Japan

Non-alcoholic fatty liver disease (NAFLD) is increasingly diagnosed worldwide and is the most common cause of abnormal liver function tests and chronic liver disease in clinical practice. Although most patients with simple steatosis will have stable disease, 10–15 % with histologically proven non-alcoholic steatohepatitis (NASH) will progress to cirrhosis and its complications such as liver failure and hepatocellular carcinoma [1–3]. A long-term follow-up study of NAFLD patients showed that patients with NASH have lower survival rates compared to patients with simple steatosis, and most of the patients in the follow-up period developed type 2 diabetes or impaired glucose tolerance [2]. Cirrhosis as a result of NAFLD is predicted to surpass chronic hepatitis C (CHC) as the leading indication for liver transplantation in the USA within the next 5 years, as the incidence of CHC is decreasing whereas that of NAFLD is increasing [4].

Although cirrhosis is a major risk factor for hepatocellular carcinoma (HCC) development, a growing number of case reports and small patient series demonstrate that liver cancers sometimes develop in individuals with NAFLD who do not also have cirrhosis [5]. The studies published in *Hepatology International* have provided further insight into the natural history of NAFLD and HCC. In this issue, Mohamad and colleagues [6] carefully investigated the characterization of NAFLD-related HCC patients with or without cirrhosis. Thirty-six patients with NAFLD and HCC in NCL (HCC-NCL group) were identified and compared to 47 patients with NAFLD-HCC and liver cirrhosis (HCC-LC group). Liver fibrosis was not present in 55.9 % of patients in the HCC-NCL group (F0), stage 1 was present in 17.6 %, stage 2 in 8.8 % and stage 3 in 17.6 %. Lobular inflammation was present in 63.6 % of non-cirrhotic patients. Patients in the HCC-NCL were older (67.5 ± 12.3 vs. 62.7 ± 8.1 years), and less likely to be obese (52 vs. 83 %) or have type 2 diabetes (38 vs. 83 %), with *p* values <0.05 for all. More interestingly, compared with the HCC-CL group, those in the HCC-NCL group were more likely to present with a single nodule (80.6 vs. 52.2 %), larger nodule size (>5 cm) (77.8 vs. 10.6 %), and receive hepatic resection as the modality of HCC treatment (66.7 vs. 17 %), and were less likely to receive loco-regional therapy (22.3 vs. 61.7 %) or orthotopic liver transplantation (OLT) (0 vs. 72.3 %), with *p* values <0.001 for all. These patients presented with larger tumors possibly because they did not receive regular medical check-ups due to their relatively preserved liver function. We have previously reported similar tumor characteristics in elderly HCC patients who also tended to have tumor markers with high DCP and low AFP [7, 8]. These results suggested that there might be a relationship between aging and HCC. Studying differences in tumor markers might be helpful in further understanding the tumor biology.

The authors also analyzed the prognosis of patients and found 86 % of patients without cirrhosis had HCC recurrence compared to only 14 % in patients with cirrhosis (*p* < 0.001). One possible explanation is that these solitary tumors in the HCC-NCL group might have an aggressive tumor biology, although this result should be interpreted carefully because more cirrhotic patients might have undergone OLT and therefore presented with fewer recurrences than non-cirrhotic patients who might have undergone resection. Whether the recurrent tumor was intrahepatic metastases or de novo recurrence could be further investigated in pathological or genetic analysis. The HCC-NCL group was less likely to have obesity, type 2 diabetes and metabolic syndrome compared to the HCC-CL group, consistent with reports of the clinical features of NAFLD and HCC [9]. The metabolic syndrome along with obesity and insulin resistance support several unique mechanisms that promote tumorigenesis. Understanding these pathways may have significant implications for pathogenesis and treatment and compel further investigation [10, 11]. Unadjusted analysis indicates that non-cirrhotics had worse survival with a mortality rate of 47 vs. 28 % in the CL group (*p* = 0.03); however, this difference in survival between the two groups was not significant after adjusting for age or OLT (*p* > 0.05).

Although provocative, these data need to be treated with caution, as there are clear limitations in the study. Since these patients were not studied as a longitudinal cohort prior to HCC treatment, there is distinct selection bias. Patients with rapid progress of fibrosis and HCC may have died before the diagnosis. In addition, regarding the metabolic syndrome, poorly controlled patients may have died from other causes before HCC development. It is hard to evaluate the onset or duration of NAFLD; however, it would be of great interest to know the duration of the disease, specifically in F0 patients with HCC. Finally, since almost half of the patients underwent OLT, the survival rate does not reflect the true natural history of the disease. The survival curve did not decline over time and OLT could be beneficial for improving liver function and reducing the possibility of de novo recurrence of HCC. Longer follow-up data will be of interest.

Despite its inherent limitations, this manuscript suggests the nature of HCC arising in the context of NAFLD may be different from that arising from other forms of liver disease. As we are in the midst of an international epidemic of NAFLD and its major comorbidities, it is critical that we understand the implications of NAFLD for HCC. This knowledge will be necessary to develop optimal screening protocols, including risk scoring systems and treatment options: new rules of engagement may be required.

## References

[1] Matteoni CA, Younossi ZM, Gramlich T, Boparai N, Liu YC, McCullough AJ. Nonalcoholic fatty liver disease: a spectrum of clinical and pathological severity. Gastroenterology 1999;116:1413–1419 10.1016/s0016-5085(99)70506-8 10348825

[2] Ekstedt M, Franzen LE, Mathiesen UL, Thorelius L, Holmqvist M, Bodemar G, Kechagias S. Long-term follow-up of patients with NAFLD and elevated liver enzymes. Hepatology 2006;44:865–873 10.1002/hep.21327 17006923

[3] Adams LA, Lymp JF, St Sauver J, Sanderson SO, Lindor KD, Feldstein A, Angulo P. The natural history of nonalcoholic fatty liver disease: a population-based cohort study. Gastroenterology 2005;129:113–121 10.1053/j.gastro.2005.04.014 16012941

[4] Holmberg SD, Spradling PR, Moorman AC, Denniston MM. Hepatitis C in the United States. N Engl J Med 2013;368:1859–1861 10.1056/NEJMp1302973 PMC5672915 23675657

[5] Sorensen HT, Mellemkjaer L, Jepsen P, Thulstrup AM, Baron J, Olsen JH, Vilstrup H. Risk of cancer in patients hospitalized with fatty liver: a Danish cohort study. J Clin Gastroenterol 2003;36:356–359 10.1097/00004836-200304000-00015 12642745

[6] Mohamad B, Shah V, Onyshchenko M, Elshamy M, Aucejo F, Lopez R, Hanouneh IA, Alhaddad R, Alkhouri N. Characterization of hepatocellular carcinoma (HCC) in non-alcoholic fatty liver disease (NAFLD) patients without cirrhosis. Hepatol Int 2015. 10.1007/s12072-015-9679-0 26558795

[7] Hamamura K, Shiratori Y, Shiina S, Imamura M, Obi S, Sato S, Yoshida H, Omata M. Unique clinical characteristics of patients with hepatocellular carcinoma who present with high plasma des-gamma-carboxy prothrombin and low serum alpha-fetoprotein. Cancer 2000;88:1557–1564 10.1002/(sici)1097-0142(20000401)88:7<1557::aid-cncr9>3.0.co;2-g 10738213

[8] Fujishima T, Ishikawa T, Shiratori Y, Kanda M, Tateishi R, Akamatsu M, Koike Y, Sato S, Obi S, Hamamura K, Teratani T, Shiina S, Yoshida H, Kawabe T, Omata M. Age-related comparison of the profiles of patients with hepatocellular carcinoma. Hepatogastroenterology 2006;53:913–918 17153452

[9] Duan XY, Qiao L, Fan JG. Clinical features of nonalcoholic fatty liver disease-associated hepatocellular carcinoma. Hepatobiliary Pancreat Dis Int 2012;11:18–27 10.1016/s1499-3872(11)60120-3 22251466

[10] Scalera A, Tarantino G. Could metabolic syndrome lead to hepatocarcinoma via non-alcoholic fatty liver disease? World J Gastroenterol 2014;20:9217–9228 10.3748/wjg.v20.i28.9217 PMC4110551 25071314

[11] Perumpail RB, Liu A, Wong RJ, Ahmed A, Harrison SA. Pathogenesis of hepatocarcinogenesis in non-cirrhotic nonalcoholic fatty liver disease: Potential mechanistic pathways. World J Hepatol 2015;7:2384–2388 10.4254/wjh.v7.i22.2384 PMC4598608 26464753

